# CD11b as a diagnostic biomarker of rheumatoid arthritis and as a risk factor of periodontitis: a pilot study

**DOI:** 10.1007/s10266-025-01129-x

**Published:** 2025-06-13

**Authors:** Marco Bonilla, Manuel Bravo, Mahtab Moradi, Natividad Martín-Morales, Enrique Raya-Álvarez, Francisco Mesa

**Affiliations:** 1 Higher Technician in Clinical, Biomedical Laboratory, Center for Biomedical Research (CIBM), University of Granada, 18016 Granada, Spain; 2 Department of Preventive and Community Dentistry, School of Dentistry, University of Granada, 18071 Granada, Spain; 3 School of Dentistry, University of Granada, 18071 Granada, Spain; 4 Department of Pathology, School of Medicine, University of Granada, 18016 Granada, Spain; 5 Department of Medicine, School of Medicine, University of Granada, 18016 Granada, Spain; 6 Department of Periodontics, School of Dentistry, University of Granada, 18071 Granada, Spain; 7 Institute of Biosanitary (IBS Institute), 18012 Granada, Spain; 8 Scientific Instrumentation Center (CIC), University of Granada, 18071 Granada, Spain; 9 Department of Rheumatology, San Cecilio University Clinical Hospital, 18016 Granada, Spain

**Keywords:** Periodontitis, Rheumatoid arthritis, Bone resorption, Flow cytometry

## Abstract

This study aimed to explore the role of CD11b, CD38, and HLA-DR in saliva, linking rheumatoid arthritis (RA) and periodontitis. We hypothesized that their expression reflects shared immunological pathways and correlates with clinical periodontal variables. An observational case–control study was conducted with RA patients (cases), healthy participants, and individuals with degenerative chronic joint pain (controls). Salivary CD11b, CD38, and HLA-DR levels were measured using flow cytometry for the first time. Periodontal assessments included PPD, in a periodontal lesion severity index (PIRIM), CAL, in a periodontal extension index (Arbes), plaque index (PI) and BOP. Biochemical markers such as anti-citrullinated peptide antibodies (ACPA), rheumatoid factor, and C-reactive protein were measured in the patients’ clinical analyses and included in this pilot study. Thirty-three RA patients and 22 controls were analyzed. CD11b (p = 0.043) and CD38 (p = 0.002) were significantly elevated in RA patients. CD11b correlated positively with BOP (p = 0.047), PI (p = 0.035), and the PIRIM index (p = 0.040). ACPA levels associated with BOP (p = 0.046), and HLA-DR levels positively associated with the number of teeth (p = 0.037). Patients with RA exhibit higher salivary levels of CD11b and CD38 compared to controls. CD11b was associated with all clinical periodontal variables except for the number of teeth. We hypothesize that CD11b acts as a key regulator of osteoclastogenesis, supporting the hypothesis of a bidirectional relationship between RA and periodontitis.

## Introduction

Rheumatoid arthritis (RA) is the most common autoimmune inflammatory rheumatic disease, primarily affecting joints through chronic synovitis that leads to bone and cartilage destruction [1]. The early production of anti-citrullinated peptide antibodies (ACPA) is a key diagnostic marker, providing significant predictive value and correlating with disease severity [2]. Although the mechanisms linking periodontitis and RA remain under investigation, extensive evidence suggests a strong connection between the two. Both diseases share common immunoinflammatory processes, including the activation of pyroptosis-related genes [3] and chronic immune dysregulation driven by microbial dysbiosis.

Periodontitis is a chronic inflammatory disease with multifactorial origins, often driven by a dysbiotic subgingival microbiome. This microbial imbalance results in the production of virulence factors, such as lipopolysaccharides, which cause immune system disruption and cytokine storms, leading to irreversible damage to the alveolar bone and periodontal ligament [4]. Notably, periodontal bacteria such as *Aggregatibacter actinomycetemcomitans* (*A. actinomycetemcomitans*) and *Porphyromonas gingivalis* (*P. gingivalis*) have been shown to trigger immune responses that promote the production of ACPA [5].

*P. gingivalis*, a member of Socransky’s red complex, plays a key role in RA due to its ability to produce peptidylarginine deiminase, which generates citrullinated peptides that contribute to the formation of ACPAs [6, 7]. Similarly, *A. actinomycetemcomitans* induces hyperactivity of human deiminases [8]. These bacteria may contribute to the pathogenesis of RA through mechanisms of molecular mimicry and immune dysregulation, further linking oral health with systemic autoimmune diseases.

Recent research has identified two novel cluster differentiation markers, CD38 and CD11b, which are measurable via flow cytometry and may serve as valuable diagnostic tools for RA due to their association with the ACPA E4 subtype [9]. CD38 is elevated in RA, but its exact role in autoimmune immunity is still debated [10]. Combined analysis of CD38 and CD11b has been shown to correlate with active disease phases and may facilitate earlier RA diagnosis, particularly during synovitis stages [11].

CD11b expression is closely associated with RA symptoms, decreasing in response to corticosteroid and anti-inflammatory treatment [12]. Cytokines such as IL-2, IL-4, TNF-α, and TNF-β trigger CD11b expression on monocytes [13], promoting the shift to M1 macrophages [14], which accumulate in the joints and contribute to inflammation [15]. Studies have also reported increased levels intraoral CD11b in patients with periodontitis [16]. However, the impact of the association between periodontitis and RA on these markers has not been fully explored.

B cells and macrophages are crucial in the autoimmune response in RA, especially after ACPA secretion [17]. Both cell types exhibit increased expression of CD38 and CD11b, and their role in chronic inflammation is significant. Additionally, the identification of HLA-DR+ antigen-presenting cells through flow cytometry allows for deeper analysis of these immune cells during inflammation [18]. Elevated levels of ACPA and rheumatoid factor (RF) in the blood correlate with increased disease activity and a poorer prognosis in RA [19].

While previous studies have explored the systemic expression of CD38 and CD11b in peripheral blood and synovial tissues of RA patients, their expression in both RA and periodontitis patients remains largely unexplored. Saliva, as a non-invasive diagnostic fluid, offers a promising alternative for monitoring immune activation in systemic and oral inflammatory diseases. Given the oral origin of immune stimuli in periodontitis and the established systemic implications of RA, assessing these markers in saliva could provide novel insights into the local expression of systemic inflammation. This approach may help identify accessible biomarkers reflective of shared pathogenic pathways, offering new avenues for early diagnosis, disease monitoring, and therapeutic stratification in patients affected by both conditions.

We hypothesize that CD38- and CD11b-positive cell populations may reflect a shared immunological axis between periodontitis and RA. These patterns could unveil novel mechanisms of inflammatory interaction between the two diseases, aiding in the identification of shared biomarkers for diagnostic or prognostic purposes.

Our aim was to evaluate, for the first time, the expression of CD38 and CD11b in saliva using flow cytometry in a case–control study (RA patients and controls), as well as the association of these markers with clinical periodontal variables.

## Methods

### Study design and participants

An observational case–control study was conducted between July and December 2024. The cases consisted of patients with RA, while the controls included healthy individuals working in the department and patients with degenerative-type chronic joint pain. Both groups were recruited from the Rheumatology Department of the San Cecilio University Clinical Hospital, Granada, Spain. Matching was performed manually, ensuring that each case was paired with a control based on similar age (within a ±5-year range), gender, and smoking status.

The inclusion criteria for cases were adult patients diagnosed with RA according to the American College of Rheumatology (ACR) criteria [20]. The inclusion criteria for controls were healthy adults or those diagnosed with fibromyalgia and/or other chronic joint disorders from the same Rheumatology Department. Exclusion criteria for both groups included having received periodontal treatment in the past year, being under periodontal maintenance, edentulous, or having fewer than 10 teeth remaining, having a systemic inflammatory disease different from those studied, diabetes, immunodeficiency, or being pregnant. Controls who had taken antibiotics and/or anti-inflammatory drugs in the last two months were also excluded.

The procedures in this study were conducted in accordance with the Declaration of Helsinki, including its most recent revision in 2013. The study methodology followed the STROBE guidelines for observational studies [21], and the checklist is attached as an appendix. All participants provided verbal informed consent regarding the study. The study protocol was approved by the Research Ethics Committee (CEim) of the provincial province of Granada, Spain, under application number: SICEIA-2024-002311.

### Study variables

#### Sociodemographic variables

Age in years, gender, and tobacco consumption (cigarettes/day).

#### Patient medication

Patients with RA received standard therapeutic regimens during the study. Specifically, they were treated with Prednisone at doses ranging from 2.5 to 7.5 mg per day, Methotrexate (a conventional synthetic disease-modifying antirheumatic drug, csDMARD) administered at 10 to 22.5 mg in a single weekly dose, or Adalimumab (a biologic DMARD) at a dose of 40 mg every two weeks.

#### Periodontal variables

The number of present teeth, probing pocket depth (PPD), gingival recession, clinical attachment loss (CAL), all measured in millimeters; bleeding on probing (BOP) [22], and visible plaque index (PI) [23], both measured as percentages. Periodontitis severity was assessed using the PIRIM score (PIRIM = ∑ (di ni)/t, where “i” is the site, “d” is the PPD of the site in mm, “n” is the absolute frequency of the sites, and “t” is the number of remaining teeth) [24]. Periodontitis extension (classified as mild, moderate, or severe) was assessed according to the Arbes criteria [25], which refers to the percentage of sites with CAL ≥ 3 mm. Periodontitis was defined as the presence of detectable interdental CAL ≥ 3 mm at ≥2 non-adjacent teeth, with PPD ≥ 4 mm and bleeding on probing, according to the latest classification of the American Academy of Periodontology and the European Federation of Periodontology, published in 2018 [23]. All measurements were taken using a manual probe PCP-UNC15 (Hu-Friedy, Chicago, IL, USA) by a single examiner, who was previously calibrated and blinded. The sole examiner, M.M., was calibrated prior to the study with the reference investigator F.M. for both inter- and intra-observer measurements of PPD and CAL at the clinic of the School of Dentistry, University of Granada. The concordance results were 79% and 82%, respectively. A ±1 mm error was accepted for each measurement. In the case of partially edentulous patients, removable dentures were removed, and for fixed prostheses, only the patient’s teeth were probed; implants were not probed.

#### Biochemical variables

**Fig. 1:**
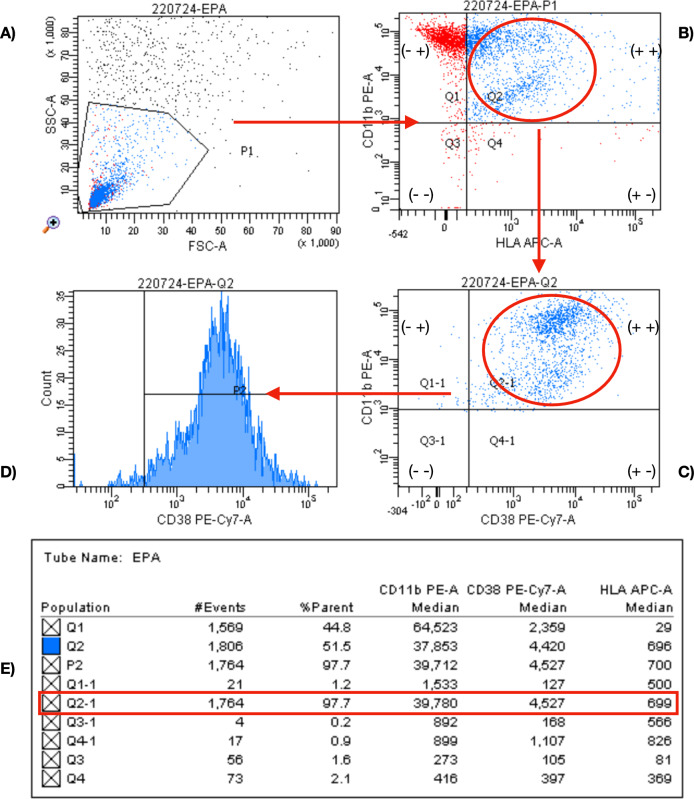
Flow cytometry analysis of HLA-DR, CD11b, and CD38 markers. **A** FSC-A vs SSC-A for identification of cell-like events. **B** Identification of HLA-DR+ and CD11b+ cells, excluding non-antigen-presenting cells. **C** Gate for selection of CD11b+ and CD38+ cells. **D** Histogram showing CD38 positivity. **E** Median fluorescence intensity (MFI) of the Q2-1 population (HLA-DR+, CD11b+, CD38+)

From the most recent laboratory tests, the following markers were measured in venous blood at the laboratory of the San Cecilio University Hospital, Granada, Spain: RF, expressed in international units/ml; ACPA, expressed in units/ml; C-reactive protein (CRP), measured by ELISA, expressed in mg/L; and erythrocyte sedimentation rate (ESR), expressed in mm for the first hour. Saliva was recollected using the passive drool method [26]. The measurement of HLA-DR, CD11b, and CD38 was performed using mean fluorescence intensity (MFI). For analysis, 500 μL of saliva were centrifuged at 1500×*g* for 15 min and resuspended in 100 μL of phosphate-buffered saline (PBS; Thermo Fisher Scientific, Waltham, MA, USA). The following antibodies were then added according to the manufacturer’s instructions: HLA-DR/DP Monoclonal Antibody (Clone MEM-136, APC-conjugated; Thermo Fisher Scientific), CD38 Monoclonal Antibody (Clone HIT2, PE-Cy7 conjugated; Thermo Fisher Scientific), and CD11b Monoclonal Antibody (Clone M1/70, PE-conjugated; Thermo Fisher Scientific). After incubation, the samples were washed twice with 1X Wash Buffer, and the Reading Reagent, both obtained from a Thermo Fisher kit (Catalog No. EPX060-10009-901). Finally, the sample was resuspended in 300 μL of Reading Reagent before analysis (Fig. 1).

### Statistical method

For the statistical analysis, IBM SPSS Statistics 22.0 (IBM Corp., Armonk, NY) was used, with descriptive and analytical methods presented at the bottom of each result table. When comparing means, to decide whether to use a parametric test (t-test) or a non-parametric test (Mann–Whitney test), we first checked visually the Normality looking at the distributions. Regarding multivariate analysis, four multiple linear regression analyses were performed, considering RF, ACPA, CD11b and CD38 as dependent variables (after their logarithmic transformation due to the skewness of their distributions). As potential predictors, the variable CASE was forced into each model, and the models were built using the forward stepwise method, considering only variables with p < 0.10 in the bivariate analyses. Periodontal variables were not allowed together in the model due to collinearity between them. Finally, no periodontal variable entered the models, and, thus, we only offer the bivariate associations in Table 3.

Regarding statistical power, the sample size of the study (22 controls and 33 cases) allows the detection of standardized differences in periodontal variables of 0.8, which is considered substantial, according to Cohen’s scale [27]. For biochemical variables, which are measured in 4 to 8 controls and 7 to 29 cases (depending on the variable), those samples allow the detection of standardized differences in the range of 1.2 to 1.6, i.e. between very large (1.20) and Huge (2.0) according to Sawilowsky [28].

## Results

Thirty-three RA patients and 22 control patients were included in the study. Table 1 presents the results of the comparison of sociodemographic variables, showing that both groups were highly similar in terms of age, gender, and tobacco use. Table 2 demonstrates that RA patients exhibited higher BOP, visible plaque levels, and periodontal lesion severity (PIRIM) compared to controls. Periodontitis cases were nearly twice as common in the RA group (60% vs. 31%), with the comparison approaching statistical significance (p = 0.069). Out of the 55 participants recruited, only 15 were analyzed using flow cytometry (Table 3). In these 15 participants, in addition to the expected higher levels of RA diagnostic markers (RF and ACPA) in RA patients, CD11b and CD38 markers were also significantly elevated in RA patients (p = 0.043 and p = 0.002, respectively).

**Table 1:** Sociodemographic variables and tobacco consumption (n = 55)

Variable	Control (n = 22)	Case (n = 33)	p-value
Age (years)			0.543^a^
Range	37–84	18–73	
Mean ± sd	57 ± 10	54 ± 14	
Sex, n (%)			0.950^b^
Male	5 (22.7)	9 (27.3)	
Female	17 (77.3)	24 (72.7)	
Tobacco			0.638^c^
No	21 (95.5)	29 (87.9)	
6–10 cig/day	1 (4.5)	1 (3.0)	
10 cig/day	–	1 (3.0)	
20 cig/day	–	2 (6.1)	

^a^Student’s t-test

^b^Chi-square test with continuity correction

^c^Fisher’s exact test (two-tailed)

**Table 2:** Periodontal variables (n = 55)

Variable	Control (n = 22)		Case (n = 33)		p-value
	n		n		
No. of teeth, mean ± sd	22	24.4 ± 3.1	33	22.8 ± 5.1	0.406^a^
Bleeding index %, mean ± sd	22	6.0 ± 7.4	33	16.9 ± 17.6	<0.001^a,b^
Plaque index %, mean ± sd	22	11.7 ± 11.1	33	21.1 ± 19.5	0.025^a,c^
PIRIM, mean ± sd	22	0.39 ± 0.61	33	0.98 ± 0.96	<0.001^a,d^
Arbes %, mean ± sd	12	11.3 ± 13.4	20	21.6 ± 21.2	0.159^a^

^a^Mann–Whitney U test

^b^95%-CI of the difference (cases–control): 2.9–18.9

^c^95%-CI of the difference (cases–control): 0.2–18.6

^d^95%-CI of the difference (cases–control): 0.13–1.95

**Table 3:** Variables and biochemical markers (n = 55)

	Control (n = 22)		Case (n = 33)		
Variable	n	mean ± sd	n	mean ± sd	p-value^a^
RF (IU/ml)	6	18 ± 1.0	19	85 ± 149	0.002
ACPA (U/ml)	4	1.2 ± 0.7	17	113.4 ± 139.0	<0.001
ERS (mm)	5	18.2 ± 9.9	29	13.0 ± 7.9	0.239
CRP (mg/L)	8	6.1 ± 6.4	29	8.1 ± 16.0	0.851
CD11b (MFI)	8	28,082 ± 22,882	7	45,088 ± 7394	0.043
CD38 (MFI)	8	1973 ± 1373	7	7444 ± 4345	0.002
HLA-DR (MFI)	8	1102 ± 959	7	688 ± 321	0.237

^a^Student’s t-test after Log_10_ transformation of variables. *RF* Rheumatoid Factor, *ACPA* anti-citrullinated peptide antibody, *ESR* erythrocyte sedimentation rate, *CRP* C-reactive protein, *MFI* Mean Fluoriscence Intensity

Table 4 presents the associations, using Pearson correlations, between quantitative periodontal clinical variables and biochemical markers. Notable findings include a positive association between RF and both BOP and PIRIM (r = 0.49, p = 0.013; r = 0.39, p = 0.052, respectively), as well as between ACPA and BOP (r = 0.44, p = 0.046). CD11b showed positive associations with all periodontal variables except for the number of present teeth. For the number of teeth, the only biochemical marker with a statistically significant association was HLA-DR (r = 0.54, p = 0.037). CRP was near statistical significance in its association with periodontitis (p = 0.060).

**Table 4:** Pearson correlations between biochemical markers (Log_10_ transformed) and periodontal variables

Periodontal variables	Biochemical markers						
	RF	ACPA	ESR	CRP	CD11b	CD38	HLA-DR
No. of teeth	n = 25	n = 21	n = 34	n = 37	n = 15	n = 15	n = 15
	r = 0.18	r = 0.34	r = 0.09	r = −0.23	r = −0.35	r = −0.10	r = 0.54
	p = 0.398	p = 0.130	p = 0.620	p = 0.165	p = 0.198	p = 0.736	p = 0.037
BOP	n = 25	n = 21	n = 34	n = 37	n = 15	n = 15	n = 15
	r = 0.49	r = 0.44	r = −0.01	r = 0.09	r = 0.52	r = 0.24	r = −0.37
	p = 0.013	p = 0.046	p = 0.962	p = 0.599	p = 0.047	p = 0.381	p = 0.170
PI	n = 25	n = 21	n = 34	n = 37	n = 15	n = 15	n = 15
	r = 0.36	r = 0.19	r = −0.10	r = 0.05	r = 0.55	r = 0.31	r = −0.37
	p = 0.077	p = 0.400	p = 0.573	p = 0.771	p = 0.035	p = 0.256	p = 0.173
PIRIM	n = 25	n = 21	n = 34	n = 37	n = 15	n = 15	n = 15
	r = 0.39	r = 0.03	r = −0.02	r = 0.04	r = 0.53	r = 0.24	r = −0.30
	p = 0.052	p = 0.913	p = 0.923	p = 0.793	p = 0.040	p = 0.387	p = 0.277
Arbes	n = 15	n = 13	n = 19	n = 20	n = 15	n = 15	n = 15
	r = 0.37	r = −0.36	r = 0.06	r = −0.04	r = 0.49	r = 0.19	r = −0.38
	p = 0.175	p = 0.232	p = 0.823	p = 0.858	p = 0.062	p = 0.496	p = 0.168

*BOP* bleeding on probing, *PI* plaque index, *RF* rheumatoid factor, *ACPA* anti-citrullinated peptide antibody, *ESR* erythrocyte sedimentation rate, *CRP* C-reactive protein

Each correlation is based on available data. Since biochemical data are missing in some patients, this explains the different sample sizes (n) found in the Table

## Discussion

To the best of our knowledge, our pilot study is the first to quantify CD11b and CD38 levels in saliva using flow cytometry in the context of RA and their relationship with periodontal indicators. Apart from our study, only two others have combined these markers for RA diagnosis [9, 11]. Our findings reveal statistically significant differences in CD11b and CD38 levels between the study groups (Table 3). CD11b demonstrated significant positive correlations with periodontitis presence (p = 0.054), BOP (p = 0.047), PI (p = 0.035), and PIRIM index (p = 0.040). Conversely, HLA-DR showed a significant positive correlation with the number of teeth (p = 0.037) (Table 4). While CD38 levels were significantly higher in RA patients (p = 0.002), this marker did not exhibit significant correlations with the periodontal indicators assessed.

CRP levels were elevated in both cases and controls. A possible explanation for this finding is the inclusion of periodontitis patients in the control group. Scientific evidence indicates that the inflammatory nature of periodontitis contributes to systemic increases in CRP levels [29].

It is noteworthy that despite observing a relatively low bleeding index in RA patients, six participants in the study were smokers, four of whom smoked fewer than five cigarettes per day. Since smoking is associated with reduced BOP [30], the actual BOP values may be underestimated. However, this effect could be offset by the proinflammatory impact of smoking on cytokines [31], which might manifest at the molecular level, even if clinical signs do not indicate pronounced inflammation.

The relationship between CD11b and BOP may be influenced by proinflammatory cytokines such as TNF-α, which are elevated in RA [32]. TNF-α participates in inflammatory signaling pathways, the activation of apoptosis signal-regulating kinase 1 (ASK1), which affects endothelial integrity [33] and facilitates leukocyte infiltration [34]. Monocytes expressing HLA-DR contribute to antigen presentation and immune activation [18], and activated macrophages can release cytokines such as IL-1β, promoting local inflammation and tissue degradation in periodontitis [35]. These processes coincide with increased expression of P-selectin, ICAM-1, and VCAM-1 [36]. In this context, elevated CD11b expression in macrophages could reflect enhanced inflammatory activation. Our findings show an association between CD11b and BOP, alongside a higher prevalence of periodontitis in RA cases (60%) compared to controls (31%). We also found a positive correlation between CD11b and PI.

**Fig. 2:**
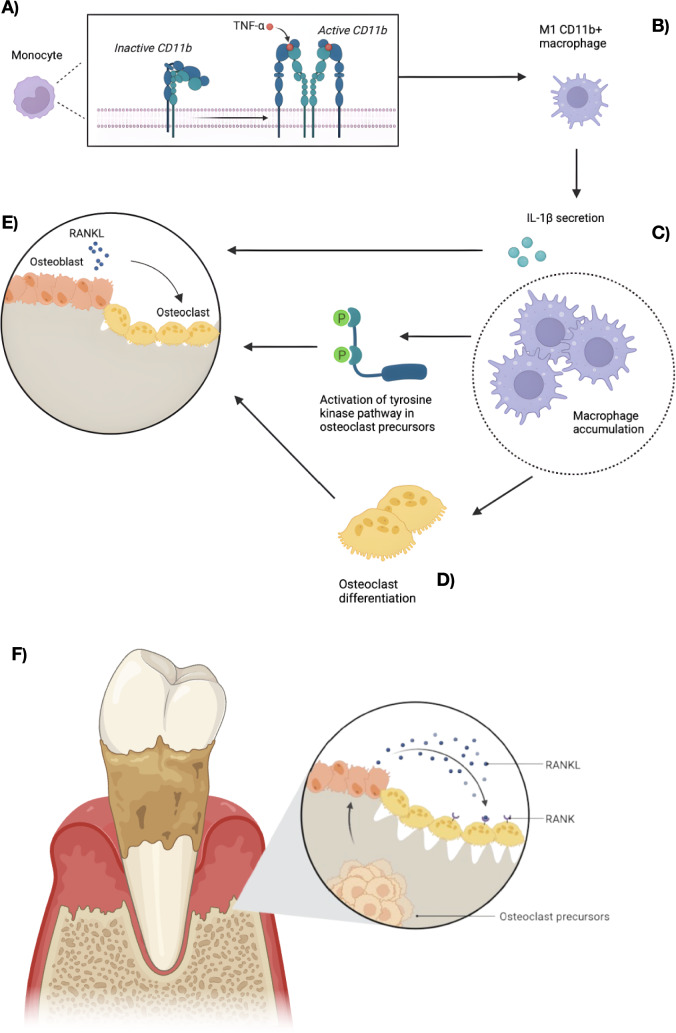
Influence of rheumatoid arthritis on periodontitis. **A** Monocytes express the integrin CD11b, which is activated by TNF-α originating from the inflammatory process [13]. **B** CD11b activation induces monocyte differentiation into M1 macrophages [14], characterized by a pro-inflammatory profile and the production of IL-1β [34]. **C** CD11b enhances macrophage recruitment to the inflammatory site [15], amplifying the local inflammatory response. **D** CD11b activation stimulates macrophage differentiation into osteoclasts via signaling pathways such as tyrosine kinase in osteoclast precursors [36]. **E** IL-1β secreted by CD11b-activated M1 macrophages, along with the activation of tyrosine kinase pathways, increases RANKL release by osteoblasts [37]. This process activates both pre-existing and newly differentiated osteoclasts. **F** The combined effects of these pathways result in increased bone resorption and osteoclast numbers mediated by CD11b: • IL-1β production: secreted by M1 macrophages differentiated via CD11b, it amplifies the inflammatory response and promotes osteoclast activation. • Tyrosine kinase pathway activation: enhanced in osteoclast precursors, it facilitates their differentiation into functional osteoclasts. • Increased macrophage recruitment: CD11b intensifies macrophage attraction to the inflammatory site, resulting in a higher number of macrophages that, mediated by CD11b, differentiate into osteoclasts, thereby increasing their total count

CD11b was significantly associated with periodontitis and its severity, as measured by the PIRIM index, suggesting that heightened CD11b expression translates into greater periodontal damage. Studies have shown that CD11b promotes osteoclastogenesis by activating the tyrosine kinase pathway, c-Fos, NFATc1 levels, and Erk activity [37]. Both interleukin-1β and the activation of the tyrosine kinase pathway converge to enhance RANKL expression and contribute to bone resorption [38]. Although these mechanisms have been primarily described in experimental settings, they may help explain the link between CD11b expression and periodontal damage in inflammatory conditions. Figure 2 in the manuscript illustrates these pathways and their potential involvement in disease-related bone loss.

Furthermore, studies in inflammatory models, such as those induced by LPS, have demonstrated that CD11b+ cells from the bone marrow and spleen can differentiate into osteoclasts [39]. Moreover, Erk1 phosphorylation promotes an increase in osteoclast numbers and resorptive activity [40]. In addition, Kim et al. demonstrated elevated CD11b levels in the peripheral blood of mice with periodontitis [16], providing additional support for its role in inflammation-associated bone alterations.

Our group, in a recently published systematic review, highlighted the citrullinating potential of two periodontopathogens, *P. gingivalis* and *A. actinomycetemcomitans* [41]. The citrullination of α-enolase by these bacteria generates RA-specific immunodominant epitopes, which could potentially induce an autoimmune response through cross-reactivity, given the similarity to bacterial enolase [42]. Furthermore, *P. gingivalis* has been shown to induce ACPA formation [43], suggesting a possible role for periodontitis in influencing the development or exacerbation of RA. Conversely, the increased CD11b levels observed in RA patients could reflect an influence on periodontitis (Fig. 2), as CD11b facilitates osteoclastogenesis, monocyte accumulation, and osteoclastic activity. This mechanism could explain the greater severity of periodontitis seen in patients with both RA and periodontitis. In our study, periodontal patients in the RA group had worse clinical outcomes compared to periodontal patients in the control group (data not shown), supporting a bidirectional relationship between the two conditions.

It is important to note that most RA patients in our study were receiving corticosteroids or other immunomodulators, which are known to reduce CD11b expression [12]. Despite this, CD11b levels were still significantly higher in RA compared to the control group, suggesting that these values could have been higher in the absence of treatment.

In our study, HLA-DR showed a statistically significant positive correlation with the number of teeth. HLA-DR plays a key role in the immune response, being involved in the recognition of antigens from periodontal pathogens [44]. We hypothesize that higher HLA-DR activity could contribute to a more efficient immune response, potentially protecting against tooth loss. Supporting this, our results showed higher HLA-DR levels and a greater number of teeth in controls, although these differences were not statistically significant compared to cases. However, BOP and PIRIM, indicative of inflammatory lesions, were significantly lower in controls.

Ultimately, RA patients express higher levels of CD11b, which is associated with increased osteoclastogenic activity and bone resorption. This finding is particularly relevant in the context of periodontitis, where RA patients with periodontitis exhibit more severe periodontal lesions compared to non-RA patients with periodontitis. Our results support the hypothesis of a bidirectional relationship between RA and periodontitis, suggesting that RA can influence periodontitis through mechanisms like those previously described, while periodontitis may act as a risk factor for RA by enhancing bone resorption through increased CD11b-levels. These findings pave the way for future research and potential therapeutic strategies.

The determination of these markers in saliva for the first time, coupled with the use of flow cytometry as a highly sensitive quantification technique is another strength of our study. Additionally, the matching of cases and controls minimized confounding factors, ensuring comparable baseline characteristics between the groups. However, due to the exploratory nature of our research (pilot study), only 15 out of the 55 participants were analyzed using flow cytometry, which limits the generalizability of our findings. Further research is needed to better understand the bidirectional relationship between RA and periodontitis.

## Conclusion

Patients with RA exhibit higher salivary levels of CD11b and CD38 compared to controls. CD11b was associated with all clinical periodontal variables except for the number of teeth. We hypothesize that CD11b acts as a key regulator of osteoclastogenesis, supporting the hypothesis of a bidirectional relationship between RA and periodontitis. 
